# Preparation of Fe_2_O_3_-Clorprenaline/Tetraphenylborate Nanospheres and Their Application as Ion Selective Electrode for Determination of Clorprenaline in Pork

**DOI:** 10.1186/s11671-016-1388-7

**Published:** 2016-04-05

**Authors:** Xintian Shao, Jing Zhang, Donghui Li, Jingli Yue, Zhenhua Chen

**Affiliations:** College of Pharmacy, Liaoning Medical University, Jinzhou, 121001 People’s Republic of China

**Keywords:** Clorprenaline hydrochloride, Fe_2_O_3_, Electrode, Pork samples

## Abstract

A novel modified ion selective electrode based on Fe_2_O_3_-clorprenaline/tetraphenylborate nanospheres (Fe_2_O_3_-CLPT NSs) as electroactive materials for the determination of clorprenaline hydrochloride (CLP) is described. The α-Fe_2_O_3_ nanoparticles (NPs) were prepared by hydrothermal synthesis, then self-assembled on CLP/tetraphenylborate (TPB) to form Fe_2_O_3_-CLPT NSs, which were used as a potentiometric electrode for analyte determination innovatively. The Fe_2_O_3_-CLPT NSs modified electrode exhibited a wider concentration range from 1.0 × 10^−7^ to 1.0 × 10^−1^ mol/L and a lower detection limit of 3.7 × 10^−8^ mol/L compared with unmodified electrodes. The selectivity of the modified electrode was evaluated by fixed interference method. The good performance of the modified electrode such as wide pH range (2.4–6.7), fast response time (15 s), and adequate lifetime (14 weeks) indicate the utility of the modified electrode for evaluation of CLP content in various real samples. Finally, the modified electrode was successfully employed to detect CLP in pork samples with satisfactory results. These results demonstrated the Fe_2_O_3_-CLPT NSs modified electrode to be a functional and convenient method to the field of potentiometry determination of CLP in real samples.

## Background

**Fig. 1:**
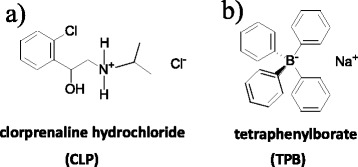
Chemical structure of clorprenaline hydrochloride (**a**) and tetraphenylborate (**b**)

Clorprenaline hydrochloride (CLP, Fig. 1a) is a type of β_2_-adrenergic agonist that is implicated in bronchial expansion and has been shown to be an effective therapeutic drug for the treatment of asthma [1, 2]. However, since previous reports have indicated that CLP can also lead to the production of leaner meat, it has been used as common feed supplements to increase feed efficiency and muscle tissue in livestock. Unfortunately, the presence of CLP residues in meat can pose significant dangers to human health, such as heart palpitations, diarrhea, muscle tremors, and even malignant tumors [3]. Due to its toxicity, the ability to detect trace amounts of CLP in food samples is highly important. Currently, several instrumental techniques have been employed for the determination of CLP in real samples, including liquid chromatography coupled with mass spectrometry (LC-MS) [4–6], gas chromatography coupled with MS (GC-MS) [7], and ion chromatography (IC) [8]. However, these techniques are time consuming and require expensive instrumentations, which is inconvenient for the routine analysis of large amounts of samples. Therefore, the development of both convenient and direct methods for the determination of CLP in real samples is urgently needed.

Potentiometric sensors were recently developed and are currently widely used in environmental monitoring, medicine detection, and biotechnology [9–11]. Potentiometric sensors possess advantages such as fast response times, simple instrumentation, ease of operation, low cost, and reasonable selectivity. For these reasons, the potentiometric sensor is a promising potential method for the determination of CLP. However, most of the reported sensors may have either of the problems such as a high detection limit or a narrow working concentration range or limited pH range. [12–14]. Therefore, finding a suitable modifier may play a crucial role in improving the detection characteristics of potentiometric sensors.

Nanomaterials have severed as promising candidates for modifiers and are commonly used in the construction of potentiometric sensors. As reported in the literatures, the linear range and detection limits of potentiometric sensors modified with nanomaterials have been improved by several orders of magnitudes compared to their unmodified counterparts [15–17]. Hematite (α-Fe_2_O_3_) is one of the most promising nanomaterials with high electric conductivity, large surface area, and good photocatalytic activity [18–20], which has received considerable attention due to its versatile applications in fabricating gas sensors [21, 22]. However, its application in constructing potentiometric sensors for analyte determination has not been reported. Thus, we present a strategy for the use of Fe_2_O_3_ nanoparticles (NPs) as modifiers of potentiometric sensors for CLP determination.

In this work, the α-Fe_2_O_3_ NPs were prepared by hydrothermal synthesis and then self-assembled on CLP/tetraphenylborate (TPB) to form Fe_2_O_3_-CLPT nanospheres (NSs). The Fe_2_O_3_-CLPT NSs were used as electroactive materials in the construction of modified electrodes. Finally, the modified electrode was applied as an indicator electrode for the convenient and rapid potentiometric determination of CLP in pork samples.

## Methods

### Reagents

In this work, all the chemical reagents were analytical grade. CLP was purchased from National Institutes for Food and Drug Control (Beijing, China), and FeCl_3_ and anhydrous CH_3_COONa were purchased from Sigma-Aldrich (St. Louis, MO, USA). Distilled water was used throughout.

### Preparation of α-Fe_2_O_3_NPs

Fe_2_O_3_ NPs were prepared by hydrothermal synthesis following the reported literature [23, 24]: a mixture of 1 mmol FeCl_3_, 3 mmol CH_3_COONa, and 50 mL distilled water was heated under stirring to form a transparent solution. Then, the obtained solution was transferred and sealed into a Teflon-lined autoclave and maintained at 180 °C for 12 h. After cooling to room temperature, the resulting red precipitate was separated by centrifuging (10,000 r/min, 10 min), repeatedly rinsed with distilled water, and finally dried at 60 °C under vacuum for further characterization and application.

### Preparation of Fe_2_O_3_-CLPT NSs

In a typical synthesis, 1 mmol Fe_2_O_3_ NPs was uniformly dispersed into a 50 mL 0.02 mol/L CLP solution after ultrasonic treatment, followed by the addition of a TPB solution (50 mL, 0.02 mol/L) into the above mixed solution drop by drop under continuous stirring. The formed precipitate was filtered with no. 3 sand core funnel and thoroughly rinsed by distilled water for 3~5 times, then stored in a desiccator until constant weight gotten. Consequently, the Fe_2_O_3_-CLPT NSs were received and used as electroactive materials of the electrode.

### Preparation of the Fe_2_O_3_-CLPT NSs Modified Graphite Electrode

A mixture containing 5 mg Fe_2_O_3_-CLPT NSs, 200 mg polyvinylchloride, 5 mL tetrahydrofuran, and 0.6 mLl dibutyl phthalate was prepared and dealt with sonicate dissolution until a clarified solution is obtained. Subsequently, the mixture was coated onto the surface of a graphite electrode, which has been polished to a mirror-like and allowed to dry under ambient condition for 2~3 days until a dried membrane formed on the surface of the electrode. Therefore, the Fe_2_O_3_-CLPT NSs modified graphite electrode for a working electrode was gotten. Before use, the electrode was soaked in a 1.0 × 10^−3^ mol/L CLP solution for 30 min to be active.

### Sample Pretreatment

The preparation of pork samples follows several reported methods [25, 26]. A 2.00-g homogenized pork sample was transferred to a 50-mL centrifuge tube and mixed with 8 mL amine acetate (0.2 mol/L, pH 5.2). After the addition of 40 μL of β-glucuronidase-arylsulfatase, the sample was incubated for 6 h at 42 °C in water bath. After being cooled down to room temperature, the mixture was vortexed for 10 min and centrifuged at 10,000 r/min for 10 min. The supernatant was transferred to another tube and mixed with 5 mL of 0.1 mol/L perchloric acid; then, the pH was adjusted to 1.0 with 1.0 mol/L perchloric acid, followed by centrifugation at 10,000 r/min for 10 min. The supernatant was transferred to another tube, and the pH was adjusted to 9.5 with 10 mol/L NaOH solution. Ten milliliter saturated NaCl solution and 10 mL isopropanol-ethyl acetate (6:4, *v*/*v*) was added, followed by vortexing for 1 min and centrifugation at 10,000 r/min for 10 min. The supernatant was collected and evaporated under a stream of nitrogen at 50 °C, after which the residue was dissolved in 10 mL HAc-NaAc buffer solution, followed by sonication for 5 min. Spiked samples were prepared in the same steps, except a known amount of CLP standard were added to the pork samples before treatment.

### Preparation of Standard CLP Solutions

A stock solution of 0.1 mol/L CLP solution was prepared using an HAc-NaAc buffer as solvent. The working solutions with concentration from 10^−1^ to 10^−9^ mol/L were completed by stepwise dilution of the stock solution.

### Characterization

**Fig. 2:**
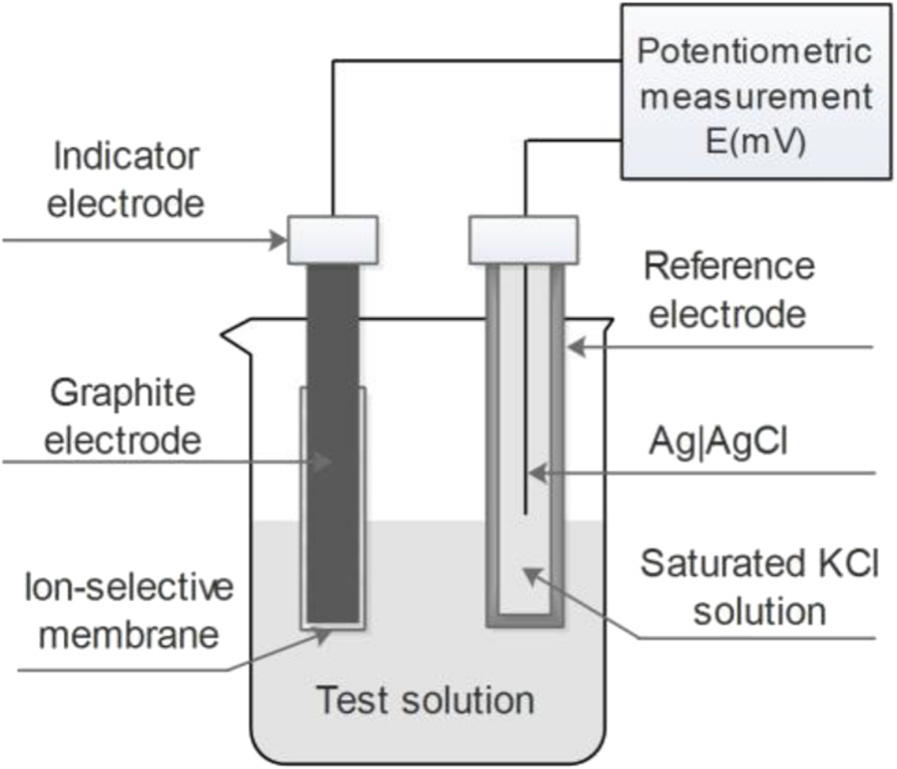
Schematic representation of the electrochemical cell

To identify the composition of the synthetic products, Fourier transform infrared spectroscopy (FTIR) was performed by using a spectrum system (SHIMADZU, Kyoto, Japan) with a resolution of 4.00 cm^−1^. The structure of the products was examined by X-ray diffraction (XRD), operating on a Rigaku Ultima IV X-ray powder diffractometer with Cu Kα radiation. The morphologies of the products were observed by scanning electron microscopy (SEM, Hitachi, S-4800, Tokyo, Japan). The electrochemical data were obtained using a CHI650D electrochemical workstation using cyclic voltammetry and electromotive force measurements (Fig. 2). A packed saturated calomel electrode (SCE) was used as an external reference. The typical cell for electrochemical data measurement was assembled as follows:

SCE, KCl (3 M) | CLP solution | the Fe_2_O_3_-CLPT NSs modified graphite electrode or the unmodified graphite electrode.

## Results and Discussion

### Characterization

**Fig. 3:**
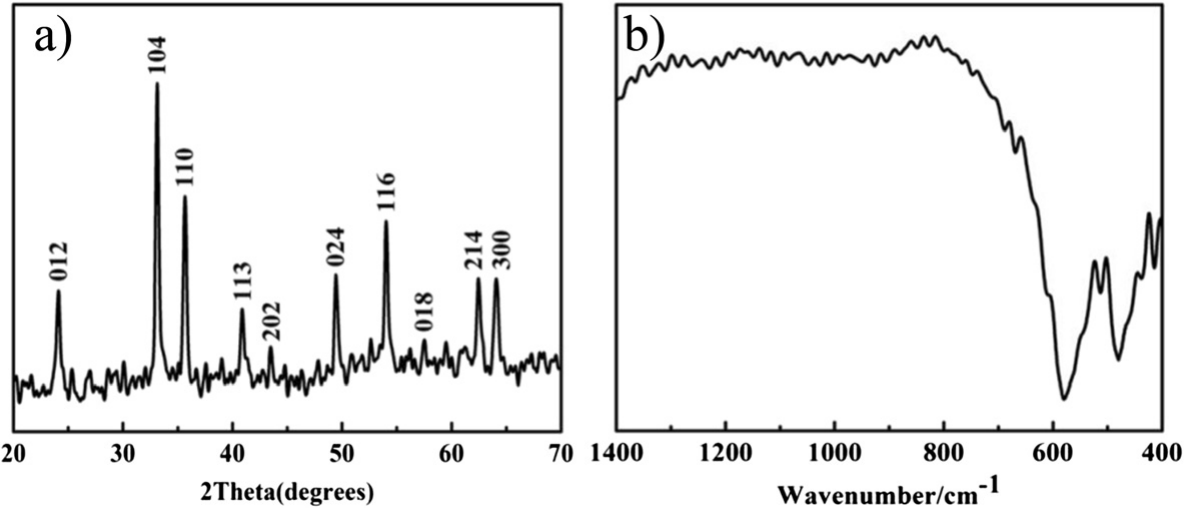
XRD pattern and FTIR spectra. XRD pattern of Fe_2_O_3_ NPs (**a**) and FTIR spectra of Fe_2_O_3_ NPs (**b**)

The composition and purity of the as-prepared Fe_2_O_3_ NPs were examined by XRD and FTIR. Figure 3a shows the XRD pattern of the prepared Fe_2_O_3_ NPs; it is evident that all of the expected peaks can be indexed to the structure of Fe_2_O_3_ NPs, which are in good agreement with the reference data (JCPDS Card No. 87-1166). Figure 3b shows the FTIR pattern of the prepared Fe_2_O_3_ NPs. At IR spectra, the band of 400–650 cm^−1^ is assigned to the stretching vibrations of the (Fe-O) band in Fe_2_O_3_ [24]. Both of the XRD and FTIR results proved that the Fe_2_O_3_ NPs were prepared successfully.

**Fig. 4:**
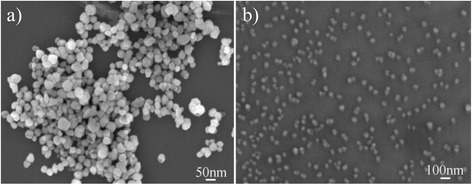
SEM images of α-Fe_2_O_3_ NPs (**a**) and Fe_2_O_3_-CLPT NSs (**b**)

The size and shape of the as-prepared samples were characterized by SEM. Figure 4a presents the SEM image of the obtained Fe_2_O_3_ NPs; it is clear that the products were irregular NPs with sizes of around 40 nm. Figure 4b shows the SEM image of the obtained Fe_2_O_3_-CLPT NSs. We can see that the NSs were well monodispersed with sizes of about 50 nm. That means the Fe_2_O_3_ NPs and Fe_2_O_3_-CLPT NSs were prepared successfully.

### Electrochemical Behaviors of Electrodes

**Fig. 5:**
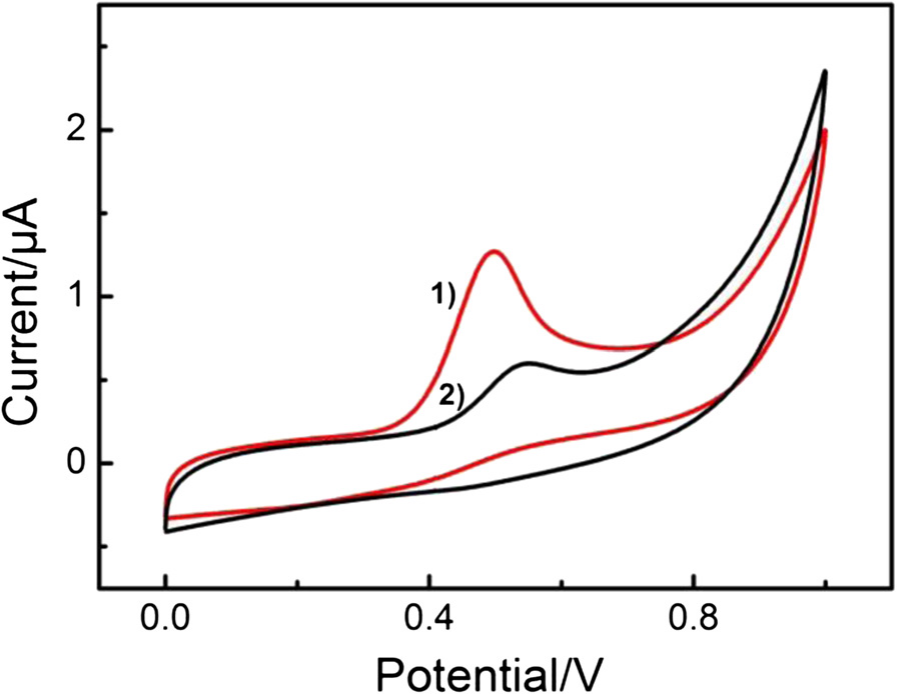
CVs of Fe_2_O_3_-CLPT NSs modified electrode (*1*) and unmodified electrode (*2*) in 1.0 × 10^−3^ mol/L CLP solution at the scan rate of 0.1 V/s

Figure 5 shows the cyclic voltammograms (CVs) of the Fe_2_O_3_-CLPT NSs modified electrode (a) and the unmodified electrode (b) in a 1.0 × 10^−3^ mol/L CLP solution at the scan rate of 0.1 V/s. Curve 1 shows that the Fe_2_O_3_-CLPT NSs modified electrode exhibited a larger anodic peak current compared to the unmodified one (curve 2), which reveals that the response of the Fe_2_O_3_-CLPT NSs modified electrode toward CLP has been improved significantly. This is most likely due to the high conductivity of Fe_2_O_3_ NPs and large surface area of Fe_2_O_3_-CLPT NSs, which promoted the electron transfer between the drug molecules.

### Calibration Graph and Measuring Range

**Fig. 6:**
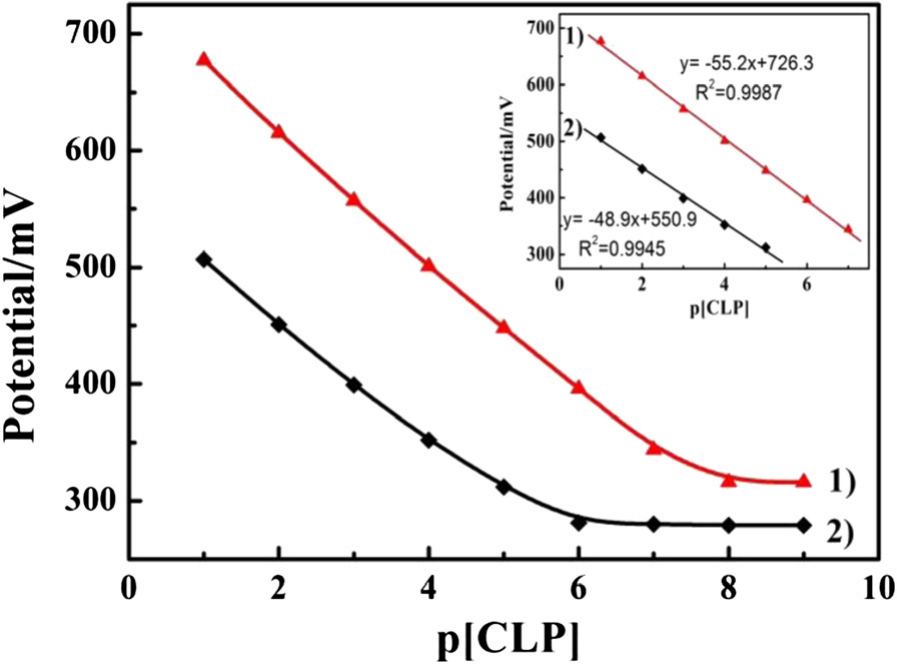
Response curves of Fe_2_O_3_-CLPT NSs modified electrode (*1*) and unmodified electrode (*2*); the *insert figure* shows their Nernstian equations

The potentiometric response of the Fe_2_O_3_-CLPT NSs modified electrode and unmodified electrode were measured in 1.0 × 10^−9^~1.0 × 10^−1^ mol/L CLP solutions. As shown in Fig. 6, the modified electrode (curve 1) displayed a better response compared with the unmodified electrode (curve 2). The results were in line with the Nernstian behavior on the electrodes, and the Nernstian equations were shown in the insert figure (Fig. 6). The concentration range of the Fe_2_O_3_-CLPT NSs modified electrode was from 1.0 × 10^−7^ to 1.0 × 10^−1^ mol/L, and the Nernstian slope was 55.2 mV/decade. The detection limit was estimated by the linearization method recommended by IUPAC. Compared to a detection limit of 3.0 × 10^−6^ mol/L for the unmodified electrode, the Fe_2_O_3_-CLPT NSs modified electrode presented a much lower detection limit of 3.7 × 10^−8^ mol/L.

### The Effect of Scan Rate on the Oxidation CLP at Electrodes

**Fig. 7:**
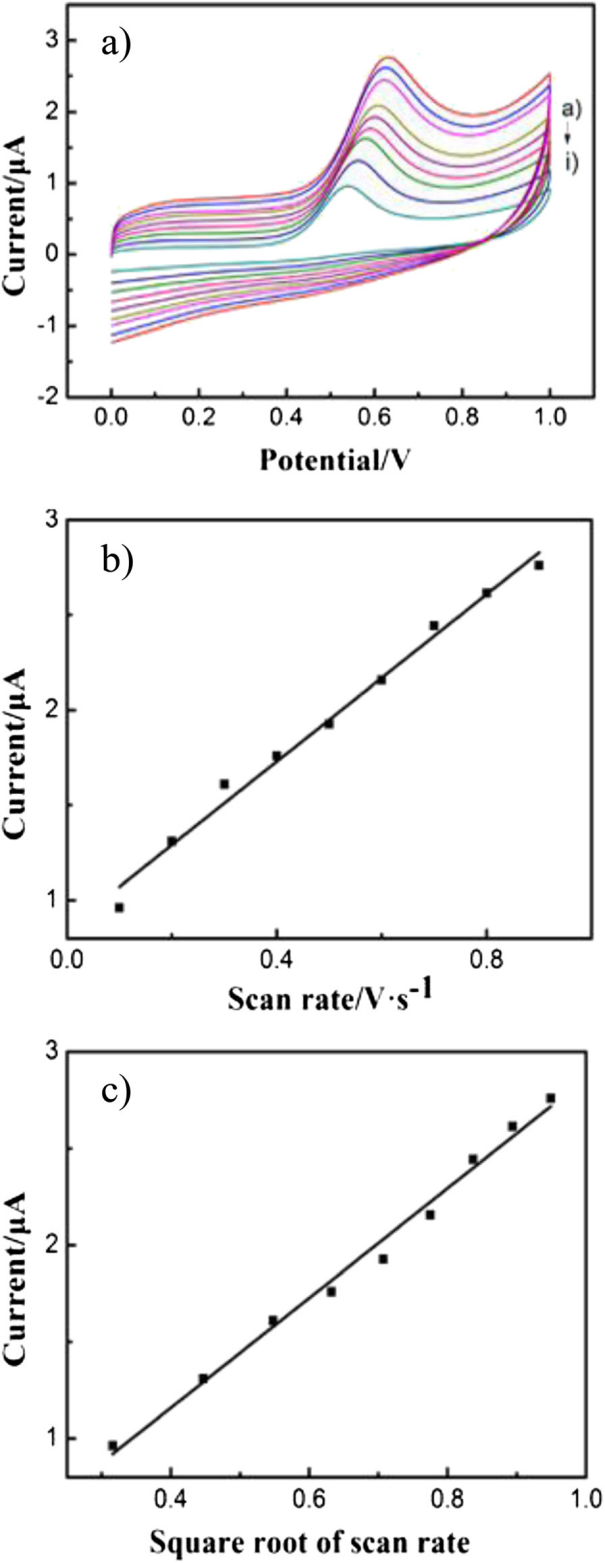
CVs of the Fe_2_O_3_-CLPT NSs modified electrode at different scan rates. CVs of the Fe_2_O_3_-CLPT NSs modified electrode in 1.0 × 10^−3^ mol/L CLP at different scan rates (*a*–*i*): 0.1, 0.2, 0.3, 0.4, 0.5, 0.6, 0.7, 0.8, and 0.9 V/s (**a**). Graph of anodic peak current versus scan rate (**b**). Graph of anodic peak current versus the square root of scan rate (**c**)

Figure 7a shows the CVs of 1.0 × 10^−3^ mol/L CLP at different scan rates in the range of 0.1~0.9 V/s at the Fe_2_O_3_-CLPT NSs modified electrode. The proportional increase of anodic peak current versus increase of scan rate is shown in Fig. 7b. The ideal correlation coefficient of 0.9877 proves that the redox reaction of CLP toward the Fe_2_O_3_-CLPT NSs modified electrode is a typical adsorption-controlled process. Figure 7c shows a linear relationship between the anodic peak current and the square root of scan rate with the correlation coefficient of 0.9890, indicating that the reaction of electron transfer is a diffusion-controlled process. Therefore, the Fe_2_O_3_-CLPT NSs modified electrode process is controlled by both processes of adsorption and diffusion simultaneously.

### Effect of pH

**Fig. 8:**
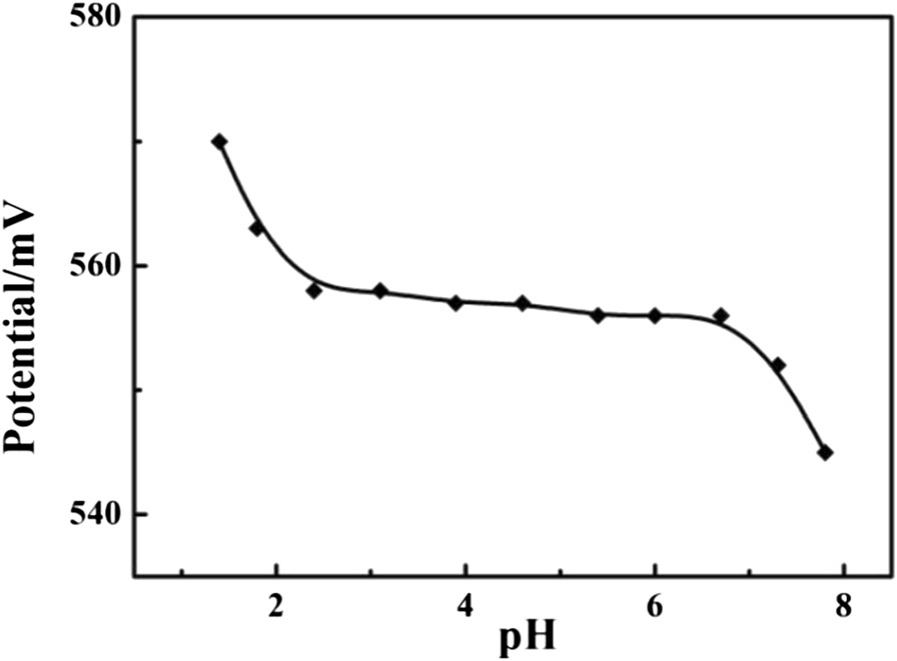
Effect of pH on the potential response of the Fe_2_O_3_-CLPT NSs modified electrode

The effect of pH on the potential response of the Fe_2_O_3_-CLPT NSs modified electrode was investigated in a 1.0 × 10^−3^ mol/L CLP solution. The pH adjustment in the solution was made with 0.01 mol/L HCl or NaOH solutions. As shown in Fig. 8, the electrode response was independent of pH in the range of 2.4 to 6.7. This range was taken as the working pH range of the modified electrode. Above this pH range, the potential showed that a sharp decrease could be attributed to the formation of deprotonation of CLP, leading to a decrease in concentration of CLP cation. Furthermore, the increase in potential below pH 2.4 is probably due to the reason that protonation of CLP was promoted at such high acidity, leading to an amount of generated CLP cation. Or electrode membrane may extract H^+^ from the medium at such lower pH and simultaneous response to H^+^ and CLP cation [27, 28].

### Response Time and Lifetime of the Modified Electrode

**Fig. 9:**
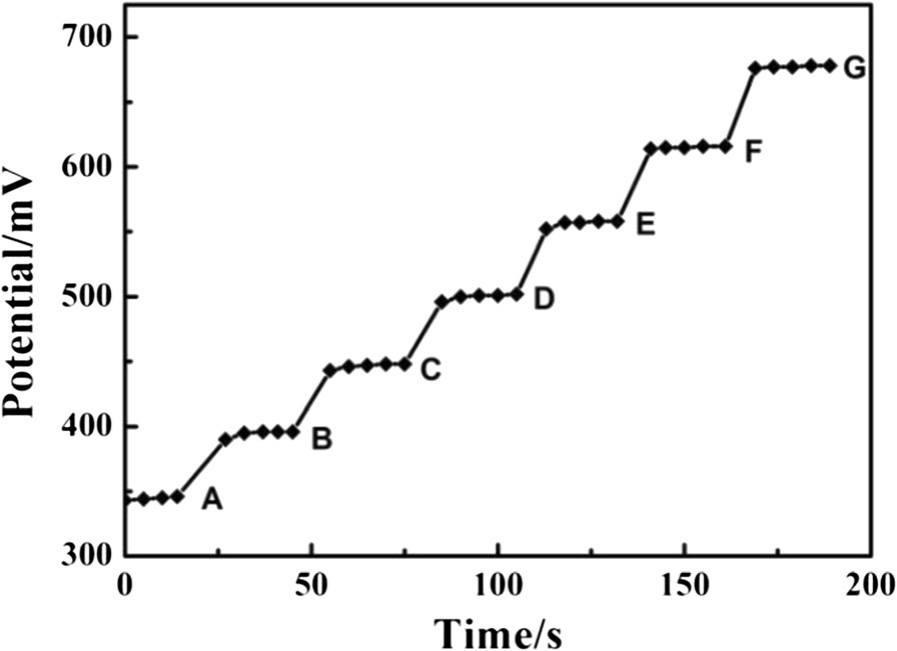
Dynamic response of the Fe_2_O_3_-CLPT NSs modified electrode obtained with step changes in concentration of CLP (*A*–*G*): 1.0 × 10^−7^, 1.0 × 10^−6^, 1.0 × 10^−5^, 1.0 × 10^−4^, 1.0 × 10^−3^, 1.0 × 10^−2^, and 1.0 × 10^−1^ mol/L

Response time is an important characteristic of an electrode. The experimental conditions—like the stirring or flow rate, the ionic concentration, composition of the text solution, and the testing temperature—all have an effect on the response time. The measurements of response time were carried out by immersing the electrode in different solutions which were tenfold different in concentration. The obtained average response time of the Fe_2_O_3_-CLPT NSs modified electrode was about 15 s at various concentrations from 1.0 × 10^−7^ to 1.0 × 10^−1^ mol/L (Fig. 9) and about 20 s in lower concentration solutions. The reproducible and stable potentials with the standard deviation of ±1.0 mV at various concentrations of CLP solutions were recorded.

**Fig. 10:**
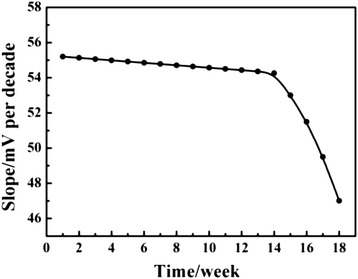
The lifetime of the Fe_2_O_3_-CLPT NSs modified electrode

In this work, the lifetime of the Fe_2_O_3_-CLPT NSs modified electrode was evaluated for a period of 18 weeks by periodically recalibrating the potentiometric response to target ion in a series of standard CLP solutions. The obtained results showed that the Fe_2_O_3_-CLPT NSs modified electrode can be used for at least 14 weeks. After this time, a gradual decrease in slope is observed as shown in Fig. 10. It is well established that the loss of CLP cation from a polymeric film due to leaching into the sample is a primary reason for the limited lifetime of the electrode.

### Selectivity of the Modified Electrode

The potentiometric selectivity coefficient (*K*_*i*,*j*_) of an ion selective electrode is commonly used as a quantitative expression of the ability of the electrode to response to primary ions in the presence of interfering ions. It was evaluated using fixed interference method (FIM) (0.01 M interfering ions and varying concentrations of target ions) in the present study. The resulting values of selectivity coefficient are provided in Table 1. Depending on this table, for all interfering ions used, the selectivity coefficients are in the order of 10^−3^ or lower proving that they would not significantly disturb the functioning of the CLP selective electrode.

**Table 1:** Selectivity coefficient values for Fe_2_O_3_-CLPT NSs modified electrode for various interfering ions

Interfering ions	*K*_*i*,*j*_
Cd^2+^	3.5 × 10^−4^
Cr^3+^	6.9 × 10^−4^
Ni^2+^	1.4 × 10^−4^
K^+^	2.6 × 10^−4^
Pb^2+^	4.5 × 10^−4^
Fe^3+^	8.3 × 10^−3^
Mn^2+^	2.7 × 10^−4^
Ag^+^	3.4 × 10^−4^
Na^+^	6.1 × 10^−4^
NH_4_^+^	9.4 × 10^−3^
Al^3+^	2.3 × 10^−5^
Fe^2+^	5.1 × 10^−4^
Hg^2+^	8.6 × 10^−3^
Co^3+^	4.2 × 10^−4^
Cu^2+^	4.6 × 10^−5^

### Reproducibility and Repeatability Stability of the Modified Electrode

The parameters of the reproducibility and repeatability were investigated in order to assess the precision of the method. For the repeatability, the relative standard deviation of ten replicate measurements with one modified electrode was 1.8 %. To evaluate the reproducibility of this modified electrode, a series of modified electrodes (six) were prepared with the same method and the responses of these modified electrodes were tested to CLP concentrations. The results showed that the standard deviation of measurements of the 1.0 × 10^−4^ mol/L CLP solution was ±2.3 mV with these six modified electrodes.

### Determination of CLP in Pork Samples

In order to confirm the sensitivity of the Fe_2_O_3_-CLPT NSs modified electrode, the developed method was applied for determination of CLP in pork samples. The pork samples were treated with the procedures described before and detected by electromotive force (EMF) measurement. No CLP was detected in the real pork samples. Then, different concentrations of CLP were spiked into the samples and detected by the Fe_2_O_3_-CLPT NSs modified electrode. The determination results are listed in Table 2.

**Table 2:** Measurement results of CLP in pork samples (*n* = 5)

Sample	Added (μmol/L)	Found (μmol/L)	Recovery (%)	RSD (%)
Pork A	1.00	0.97 ± 0.11	97.0	2.1
Pork B	5.00	4.89 ± 0.16	97.8	1.7
Pork C	10.00	9.83 ± 0.21	98.3	1.4
Pork D	20.00	19.70 ± 0.24	98.5	1.2

## Conclusions

In the present work, a new electroactive material with a nano-size and large specific surface area has been prepared and characterized using FTIR, XRD, and SEM. The obtained Fe_2_O_3_-CLPT NSs were efficiently applied for construction of CLP ion selective electrodes. Satisfactory results were received from the application of the modified electrode to the determination of CLP in pure solutions and pork samples. The modified electrode displayed a wide concentration range and a low detection limit, providing comparable optical selectivity toward CLP with better response time of 15 s. These results indicated that the Fe_2_O_3_-CLPT NSs modified electrode offered a viable technique for the determination of CLP in the real samples, with its inherent advantages of high selectivity, rapid response, simple operation, precise results, and low cost. However, compared with other instrumental techniques such as HPLC-MS, the disparity in a detection limit also existed. Therefore, further improvements on electrode performance, like reducing the detection limit and increasing the speed of response, should be the future research focuses.
